# Effects of Cocaine on Human Glial-Derived Extracellular Vesicles

**DOI:** 10.3389/fcell.2020.563441

**Published:** 2021-01-11

**Authors:** Sanjay Kumar, Qiana L. Matthews, Brian Sims

**Affiliations:** ^1^Department of Pediatrics/Division of Neonatology and Center of Glial Biology in Medicine at the University of Alabama School of Medicine, University of Alabama, Birmingham, AL, United States; ^2^Microbiology Program, Department of Biological Sciences, College of Science, Technology, Engineering and Mathematics, Alabama State University, Montgomery, AL, United States

**Keywords:** HMC3, microglia, extracellular vesicles, cocaine, brain

## Abstract

**Background:**

Microglia are important myeloid cells present in the brain parenchyma that serve a surveillance function in the central nervous system. Microglial cell activation results in neuroinflammation that, when prolonged, can disrupt immune homeostasis and neurogenesis. Activated microglia-derived extracellular vesicles (EVs) may be involved in the propagation of inflammatory responses and modulation of cell-to-cell communication. However, a complete understanding of how EVs are regulated by drugs of abuse, such as cocaine, is still lacking.

**Findings:**

Cocaine exposure reduced human microglial cell (HMC3) viability, decreased expression of CD63 and dectin-1 in HMC3-derived EVs, and increased expression of the apoptotic marker histone H2A.x in HMC3-derived EVs.

**Conclusion:**

Cocaine impacts HMC3 cell viability and specific EV protein expression, which could disrupt cellular signaling and cell-to-cell communication.

## Introduction

Microglial activation is a pivotal focus of neurobiology (Franco and Fernandez-Suarez, 2015; Liddelow et al., 2017; Regen et al., 2017). Microglia can be neuroprotective by mediating neuroinflammation and the release of various substrates (Polazzi and Monti, 2010; Chen and Trapp, 2016; Condello et al., 2018). However, this changes under various conditions (Szepesi et al., 2018). In a study looking at meningitis there appeared to be a feedback mechanism involving IL-10 and Il-6 which are anti-inflammatory and proinflammatory, respectively (Rock et al., 2004). Microglia can release trophic factors which was evident when microglia were depleted with subsequent neuronal loss. It appears that the pathological state may dictate whether the microglia is protective or not (Szepesi et al., 2018).

One pathological state that causes microglial activation is stress (Bollinger et al., 2017; Lehmann et al., 2018; Zhang et al., 2020). Stress can dictate the relationship between the nervous system and the immune system. One known stress, alcohol, is well studied and in autopsy studies of alcoholics it shows an increase in microglial activation (Dennis et al., 2014). Activation of microglia in this case causes an increase in proinflammatory substances which can cause neuronal death (Dennis et al., 2014). Chronic exposure does not induce microglial activation as much as an acute exposure (Sutherland et al., 2013). Alcohol can affect many biological processes but in a recent study it was shown that alcohol had a significant effect on both the composition and production of exosomes which can have significant physiological consequences (Crenshaw et al., 2019).

Extracellular vesicles (EVs) are small nanovesicles which can include exosomes (30–120 nm) and ectosomes (100–500 nm). EVs are released from most cell types including cells of the nervous system (Fitzner et al., 2011; Chivet et al., 2012; Wang et al., 2012; Glebov et al., 2015). The formation of EVs involves multivesicular bodies (exosomes) and plasma membrane (ectosomes). EVs are heterogeneous as it pertains to its cargo and membrane proteins. EVs contain lipids, proteins, RNA and microRNA. EVs are involved in intercellular communication and their regulation is paramount.

Another drug that is a potent activator of microglia (Burkovetskaya et al., 2020; Linker et al., 2020) and may regulate EVs is cocaine. Cocaine has been shown to increase microglial activation in rodent striatum and hippocampus (Blanco-Calvo et al., 2014). When human glioblastoma cells were treated with cocaine the release of EVs was stimulated, in particular the exosomes in a time dependent manner (Carone et al., 2015). The greatest effect was seen at the highest concentration, 150 μM, used. There is a paucity of data on what the effects of cocaine may be on EV regulation and composition in microglia. Thus, in this study we aimed to investigate what effect cocaine had on EV composition, size, and quantity in human microglia.

## Materials and Methods

### Cell Culture and Treatment

Human microglial cells (CRL-3304) were purchased from American Type Culture Collection (ATCC) (VA, United States). Human microglial cells (HMC3) were cultured in ATCC recommended Eagle’s Minimum Essential Medium (EMEM) supplemented with 11.2% Fetal Bovine Serum (FBS), 1% Pen/strep, 0.05% Amphotericin-B at 37°C in 5% CO_2_.

HMC3 microglia cells were plated at 2 × 10^6^ cells/dish and allowed to adjust overnight before cocaine dosing. The next morning, old media was removed and fresh exosome-depleted complete medium was added to each dish, Medium-only served as experimental control; however, exosome-depleted complete media containing 1 μM, and 100 μM of cocaine served as experimental treated-group which were incubated for 24 h at 37°C in 5% CO_2_. All experiments were performed either in three or five independent replicates.

### Assessment of Cell Viability

To test the percent cell viability trypan blue exclusion test was performed. For trypan blue test 5 μL of cell suspension was mixed with 45 μL of trypan blue dye and 10 μL of this cell mix was loaded to hemocytometer to perform a live/dead cell count. Cells were counted in four blocks of the hemocytometer under a light microscope and the average of the four blocks was taken and multiplied by the respective dilution factor and 10,000. The cell viability was represented in percent cell viability.

### Isolation of EVs

Isolation of microglia cell derived EVs was carried out using condition medium after 24 h of cocaine exposure at 1 μM, 100 μM, and control (No-treatment). Condition media was carefully collected and spun down at 300 × *g* [1,300 revolutions per minute (rpm)] at 4°C for 10 min using a Sorvall RT 6000 refrigerated centrifuge. The supernatant was further spun at 2,600 × *g* (3,900 rpm) at 4°C for 10 min. The supernatant was filtered with 0.22 μM membrane and tubes were balanced using a 5% sucrose solution in phosphate buffer saline (PBS) containing a 1x protease inhibitor cocktail. Ultracentrifuge tubes were spun at 20,000 × *g* (10,800 rpm) at 4°C for 45 min in an SW41T1 swinging bucket rotor at 4°C using a Beckmann Coulter Optima TML-70K Ultracentrifuge. Exosome fraction was collected after 70 min spun at 110,000 × *g* (32,000 rpm) at 4°C and were subjected to further experiments.

### Assessment of EV-Size and -Concentration

To analyze the size and concentration of HMC3 cell-derived EVs (particle per mL), nanoparticle tracking analysis (NTA) was performed using NanoSight-LM10, Malvern Instrument, Inc., Malvern, United Kingdom. The samples were prepared at a dilution of 1:100 in 1x PBS and loaded in a 0.3 mL disposable syringe. The NTA works on the principle of the Brownian movement of the particle to analyze the size and concentration of EVs in the samples. The mean values of the replicate were recorded and processed for each reading frame of the five independent experiments.

The size and morphology of exosomes analyzed using transmission EM (TEM). For TEM, fixed exosome samples were loaded on the EM-grid and incubated for 1 min at RT and immediately stained with 7 μL of filtered uranyl acetate (UA) solution on the surface of the EM-grid. After 15 s, samples were observed under TEM (Tecnai 120 kV (FEI, Hillsboro, OR, United States) at 80 kV within 24 h as compared to the negatively stained grids. Digital images were captured with a BioSprint 29 CCD Camera (AMT, Woburn, MA, United States).

### Dot Blot Analysis

To examine the expression of apoptotic markers, exosomal markers, and HSPs, dot blots analysis was carried out using 5 μg per dot total EVs-lysate after boiling at 99°C for 5 min. Membranes were allowed to dry for 5 min and non-specific binding was blocked with 1x Pierce Fast blocker for 10 min at room temperature. Membranes were incubated with the primary antibodies of CD9 (1 μg/mL), CD63 (0.5 μg/mL), cleaved caspase-9 (1:1,000), cleaved caspase-3 (1:1,000), H2A.x (1:1,000), Hsp70 (1:1,000), Hsp90-β, (1:1,000), Dectin-1 (1 μg/mL), TLR2 (1:2,000), for 1 h at room temperature. Membranes were washed three times 10 min each with 1x TBST-20 buffer containing 0.09% tween-20. HRP-conjugated appropriate secondary antibodies either goat anti-rabbit (1:1,000) or goat Anti-mouse (1:1,000 were used to incubate the membranes using 2% non-fat milk solution in TBST-20 buffer for 1 h at room temperature. Membranes were washed 3-times 10 min each with TBST-20. Targeted proteins on the membranes were detected using ECL liquid substrate system (Invitrogen, MA, United States) under a Bio-Rad ChemiDoc TM XRS+ system.

Statistical analysis: For multi group comparisons, one-way ANOVA was used. Statistical significance was established to be (^∗^) *P* < 0.05, (^∗∗^) *P* < 0.01.

## Results

**FIGURE 1:**
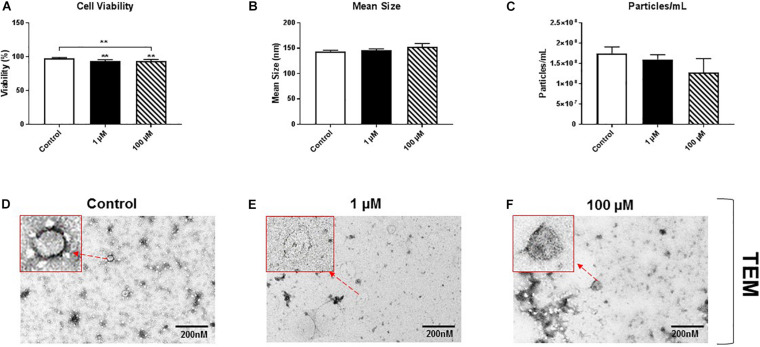
Cocaine reduces HMC3 cell viability. HMC3 cells were exposed to 1 or 100 μM cocaine for 24 h, after which cell viability (trypan blue exclusion assay) and EV size and quantity were evaluated using NTA and TEM. **(A)** Cocaine significantly reduced HMC3 cell viability when compared with the control condition. Cocaine did not alter **(B,D–F)** EV size but **(C)** slightly decreased EV production (particles per mL) compared with the control condition, although this difference was not significant. Data were obtained from 3 to 5 independent experiments performed in triplicate. Statistical significance is indicated as *P < 0.05 and **P < 0.01.

HMC3 cells, an immortalized human microglial cell line, were exposed to 1 or 100 μM cocaine, which was freshly prepared in exosome-depleted medium from 5.9 mM stock, for 24 h. Cell viability, assessed using trypan blue exclusion assay, showed a significant decrease in cell viability after exposure to 1 or 100 μM cocaine compared with the control condition (Figure 1A and Supplementary Figures 1A, 5), indicating that cocaine has a detrimental effect on microglia cells.

To test the effect of cocaine exposure on HMC3 cell-derived EVs, we cultured cells in complete Eagle’s Minimum Essential Medium using exosome-depleted fetal bovine serum with 1 or 100 μM cocaine. Medium only was used as a control. After incubating cells with cocaine, EVs released into the medium were isolated and purified using a standard ultracentrifugation procedure (Bell et al., 2019; Crenshaw et al., 2019; Jones et al., 2019). The size and quantity of isolated EVs were measured using NTA and TEM. We found that cocaine did not alter the size of EVs and EVs are visible and easily recognizable (Figure 1B and Supplementary Figures 1B,D–F), which ranged from 30 to 150 nm in diameter across conditions, consistent with previous studies (Bell et al., 2019; Crenshaw et al., 2019; Jones et al., 2019; Kumar et al., 2020). However, 100 μM cocaine slightly reduced the number of EV particles per mL compared with the 1 μM cocaine and control conditions, although this difference was not significant (Figure 1C and Supplementary Figures 1C,D–F).

**FIGURE 2:**
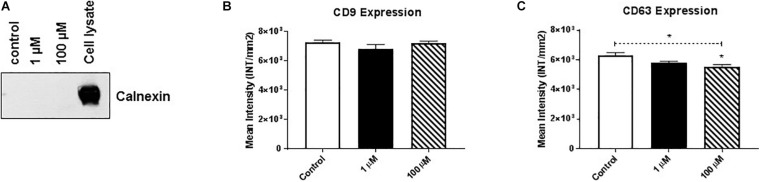
Cocaine modulates expression of EV markers in HMC3 cells. Equal amounts (5 μg/dot) of total protein from HMC3 cell-derived EVs derived after cocaine treatment with 1 or 100 μM cocaine were loaded onto nitrocellulose membranes to assess the expression of CD9 and CD63. Cocaine **(A)** demonstrated purity of exosomes **(B)** did not alter CD9 expression but **(C)** significantly decreased CD63 expression compared with the control condition. Statistical significant difference obtained from 5-independent experiment is indicated as *P < 0.05 and **P < 0.01.

Membrane-bound proteins perform a variety of vital functions for the survival of organisms, including playing critical roles in signal transduction, cell-to-cell communication, transportation, and adhesion. Therefore, using western/dot blot analysis, we evaluated the expression of select membrane-associated proteins in HMC3 cell-derived EVs that are critical for cellular signaling and are known EV-associated markers, including cluster of differentiation (CD)9 and CD63 (Polazzi and Monti, 2010; Chen and Trapp, 2016; Condello et al., 2018). Based on the evaluation of calnexin western blot analysis we found that purified exosomes (Figure 2A) after cocaine exposure did not alter CD9 expression (Figure 2B and Supplementary Figure 2B) but significantly reduced CD63 expression in EVs (Figure 2C and Supplementary Figure 2C).

**FIGURE 3:**
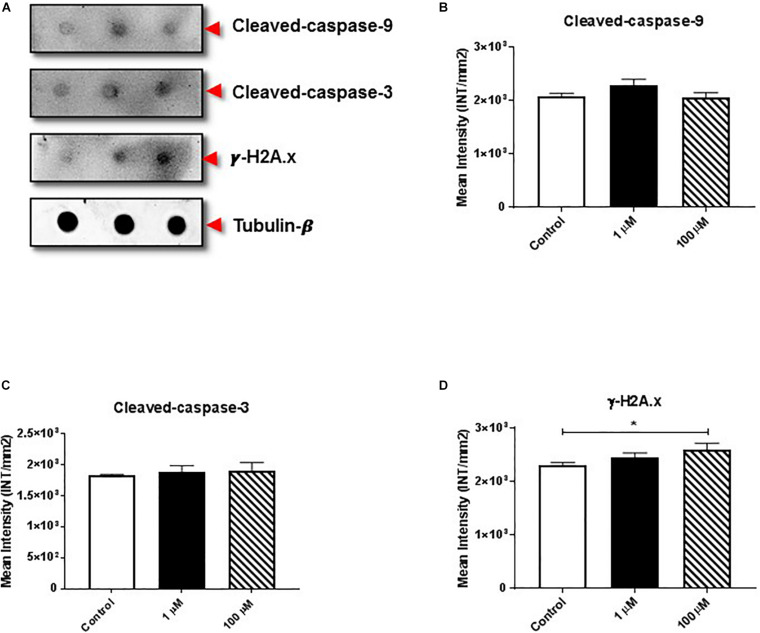
Cocaine affects expression of apoptotic markers in HMC3 cell-derived EVs. **(A)** Equal amounts (5 μg/dot) of total protein from HMC3 cell-derived EVs derived after cocaine treatment with 1 or 100 μM cocaine were loaded onto nitrocellulose membranes to assess the expression of cleaved caspase-9/-3 and histone H2A.x. Cocaine did not alter the expression of **(B)** cleaved caspase-9 or **(C)** cleaved caspase-3. However, **(D)** 100 μM cocaine significantly reduced the expression of histone H2A.x compared with the control condition. Statistical values obtained from 5-independent experiment is indicated as *P < 0.05.

The proteolytic cleavage of caspases is a unique characteristic of apoptotic cell death. We examined the effect of cocaine exposure on expression of cleaved caspase-9 and -3 along with histone H2A.x (Figure 3A), a well-known marker of double-stranded DNA break or damage (Talasz et al., 2002; Stucki, 2009; Yin et al., 2019). We found that cocaine exposure did not alter the expression of cleaved caspase-9 or -3 (Figures 3B,C and Supplementary Figures 3B,C). However, 100 μM cocaine significantly increased histone H2A.x expression (Figure 3D and Supplementary Figure 3D), supporting previous findings (Bell et al., 2019).

**FIGURE 4:**
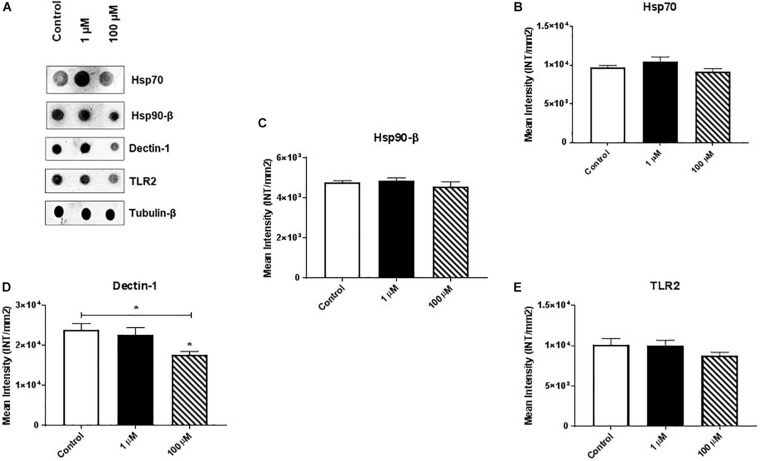
Cocaine affects expression of dectin-1 in HMC3 cell-derived EVs. Equal amounts (5 μg/dot) of total protein from HMC3 cell-derived EVs were loaded onto nitrocellulose membranes. Cocaine did not alter the expression of **(A)** representative dot blot, **(B)** Hsp70, and **(C)** Hsp90-β, or **(E)** TLR2. However, **(D)** 100 μM cocaine significantly reduced the expression of dectin-1 compared with the control condition. *P < 0.05 indicates statistical significant difference between control vs. treated.

Heat shock proteins (HSPs) are an evolutionarily conserved group of proteins expressed in all eukaryotes and some prokaryotes (Kumar et al., 2013, 2016). In particular, Hsp70 and Hsp90-β act as molecular chaperones that assist proper folding or refolding of newly synthesized polypeptide chains and thereby exert a cytoprotective effect against stress-induced apoptosis. We found that cocaine exposure had no/minimal effect on expression of Hsp70 or Hsp90-β (Figures 4A–C and Supplementary Figures 4B,C).

Finally, we measured the expression of dectin-1 and Toll-like receptor 2 (TLR2) in HMC3 cell-derived EVs. Dectin-1 is a C-type glycoprotein/lectin receptor expressed on macrophages, microglia, monocytes, dendritic cells, neutrophils, and a subset of T cells (Shah et al., 2008; Maneu et al., 2011) that plays important roles in the secretion of crucial cellular proteins (e.g., pro-regenerative factors) and anti-fungal immunity (Willment et al., 2001; Kato et al., 2006). TLR2 is a key regulator that induces activation of NF-kB and controls the expression of immune and inflammatory response-related genes (Medzhitov et al., 1997). We found that 100 μM cocaine significantly reduced the expression of dectin-1 (Figures 4A,D and Supplementary Figure 4D) but had no effect on TLR2 expression (Figure 4A,E and Supplementary Figure 4E). These results are consistent with previous reports that dectin-1 and TLR2 activation modulate macrophage function in the central nervous system and reduce the damaging effects of inflammation (Dennehy et al., 2008; Kelly et al., 2008; Lee et al., 2009; Gensel et al., 2015). Furthermore, these results support previous studies showing that cocaine activates brain microglial cells and that activated microglia-derived EVs play important roles in neuroinflammation and modulation of cell-to-cell communication (Cotto et al., 2018; Periyasamy et al., 2018).

## Discussion

Microglial cells are important cells in the CNS and are the resident macrophages. Activated microglia release many harmful substances such as free radicals which often times leads to death. The current study was designed to examine cocaine induced microglial cell death and focus on cocaine’s effect on EV proteins. The study provides novel insight into cocaine’s effect on exosome biogenesis in microglia. Prior to our work, there has been a limited study of cocaine’s effect on extracellular vesicles in the brain. Cocaine-induced EV regulation has been studied but not in microglia. Cocaine induced EV regulation involves the sigma 1 receptor (Sig-1R), ADP ribosylation factor 6 (ARF6) and myosin light chain kinase (Machado-Pineda et al., 2018) but still remains poorly understood. Cocaine self-administration was shown to alter the signaling of neuronal exosomes to astrocytes using a CD63 reporter (Jarvis et al., 2019). However, no studies have investigated the role of cocaine on extracellular vesicular proteins in human microglia. Though the study is in an immortalized cell line, and limited, it is the first attempt at understanding the role of cocaine on EVs in microglia. These data allow us to design future studies and investigate these finding in animal work.

Here we show targets of cocaine as it pertains to EV regulation. Carone et al. demonstrated that cocaine increases EV release, mainly exosomes in glioblastoma cultures. The conclusion of that work was that cocaine could exert its’ effect on through lipid rafts or potentially cocaine-induced modifications but not no evidence that cocaine alters the ESCART pathway (Carone et al., 2015). We demonstrated that cocaine does not affect some EV proteins like CD9 but decreases CD63 which are both located on exosomes. CD9 has been shown to enhance pathogenicity in HIV-1 infection (Sims et al., 2018) and be involved in cell adhesion (Machado-Pineda et al., 2018) and migration (Blake et al., 2018). CD63 is a tetraspanin involved in cell development, activation, growth and motility (Kang et al., 2014; Forte et al., 2017; Raaben et al., 2017). There was not a significant difference in CD9 but we did demonstrate a preferential effect on CD63 which may have a role in microglia activation, growth and motility. Our findings suggest that there is a decrease in CD63 but did not have a major impact on EV characteristics. It is difficult to say if a more chronic exposure to cocaine would cause a more dramatic effect. One limitation of our study was it was over 24 h and a single dose of cocaine. In a study using positron emission tomography imaging in cocaine abusers, it did not show that there was a significant difference in microglial activation when they analyzed the 18 kDa translocator protein (TSPO) (Narendran et al., 2014).

At our higher concentration, cocaine did cause a significant effect on cell viability. The dose corresponds to other concentration reported in the literature (Liao et al., 2016; Periyasamy et al., 2018). The effect of cocaine on apoptosis was investigated by examining cleaved caspase 3 and 9. Cocaine did not have any effect on the caspases examined. However, histone 2A.x, which forms when double stranded breaks appear (Johnston et al., 2015), was elevated. The results suggest that there is evidence of cell injury/death which may be time dependent and may be more dramatic with longer exposures. We also, hypothesized that since cocaine administration caused cell death the heat shock proteins would not be increased and that is revealed in this study.

In many cases, when microglia were studied TLR2 has been shown to be a target that is increased when microglia are activated (Liao et al., 2016). Thus, we speculated that we would also see the same response in the exosome fraction of TLR2 but cocaine had no effect on these levels. However, these studies were performed in BV2 microglial cells which suggests there may be differences that are cell line specific (Baldwin et al., 2015). Dectin-1 is involved in neuroinflammation and secretory cellular processes and should be affected by cocaine administration (Baldwin et al., 2015; Gensel et al., 2015; Ye et al., 2020). We demonstrated there was a decrease as predicted in the dectin-1 levels.

## Conclusion

Our findings demonstrate the impact of cocaine exposure on HMC3 cells and HMC3 cell-derived EVs. We found that cocaine exposure reduced HMC3 cell viability and has a slight downward trend in the quantity of EVs. We have not answered in this study whether a more chronic exposure could disrupt EV biogenesis in microglia. We found that cocaine exposure dose-dependently increased expression of the apoptotic marker histone H2A.x in HMC3 cell-derived EVs, suggesting that cocaine reduces cell viability. Although cocaine exposure did not affect levels of various EV biogenesis proteins [(e.g., small GTPase RAB-5, 7, 11, 27A, and 35 (data not shown)], it reduced the expression of CD63 in HMC3 cell-derived EVs. This down-regulation of CD63 and dectin-1 in HMC3 cell-derived EVs suggests that cocaine’s effects are specific to these proteins but not others examined. CD63 has a major role in EV production and endosomal sorting and a decrease in this protein may lead to altered exosome release and subsequent cell-to-cell communication. These results contrast with those found in murine-derived EVs. There may be more specie-specific differences in cocaine disruption of EV formation. More human studies are needed, and further investigations are warranted to elucidate the mechanisms involved in the interplay between cocaine and EVs.

## Data Availability Statement

The original contributions presented in the study are included in the article/Supplementary Material, further inquiries can be directed to the corresponding author/s.

## Ethics Statement

Ethics approval and consent to participate was not needed. HMC3 cells were purchased from ATCC (Manassas, VA, United States).

## Author Contributions

SK, QM, and BS contributed to experimental design and manuscript writing. SK executed the experiments. All authors contributed to the article and approved the submitted version.

## Conflict of Interest

The authors declare that the research was conducted in the absence of any commercial or financial relationships that could be construed as a potential conflict of interest.

## Supplementary Material

Supplementary Figure 1 Cocaine reduces HMC3 cell viability. HMC3 cells were exposed to 1 μM or 100 μM cocaine for 24 h, after which cell viability (trypan blue exclusion assay) and EV size and quantity were evaluated using NTA and TEM. **(A)** Cocaine significantly reduced HMC3 cell viability when compared with the control condition. Cocaine did not alter (**B** and **D–F**) EV size but **(C)** slightly decreased EV production (particles per mL) compared with the control condition, although this difference was not significant. Data were obtained from 3–5 independent experiments performed in triplicate. Statistical significance is indicated as **P* < 0.05 and ***P* < 0.01.

Supplementary Figure 2 Cocaine modulates expression of EV markers in HMC3 cells. Equal amounts (5 μg/dot) of total protein from HMC3 cell-derived EVs derived after treatment with 1 μM or 100 μM cocaine. These samples were loaded onto nitrocellulose membranes to assess the expression of CD9 and CD63. Cocaine **(A)** demonstrated purity of exosomes **(B)** did not alter CD9 expression but **(C)** significantly decreased CD63 expression compared with the control condition. Statistical significant difference obtained from 5-independent experiment is indicated as **P* < 0.05 and ***P* < 0.01.

Supplementary Figure 3 Cocaine affects expression of apoptotic markers in HMC3 cell-derived EVs. **(A)** Equal amounts (5 μg/dot) of total protein from HMC3 cell-derived EVs derived after treatment with 1 μM or 100 μM cocaine. These samples were loaded onto nitrocellulose membranesto assess the expression of cleaved caspase-9/-3 and histone H2A.x. Cocaine did not alter the expression of **(B)** cleaved caspase-9 or **(C)** cleaved caspase-3. However, **(D)** 100 μM cocaine significantly reduced the expression of histone H2A.x compared with the control condition. Statistical values obtained from 5-independent experiment is indicated as **P* < 0.05.

Supplementary Figure 4 Cocaine affects expression of dectin-1 in HMC3 cell-derived EVs. Equal amounts (5 μg/dot) of total protein from HMC3 cell-derived EVs were loaded onto nitrocellulose membranes. Cocaine did not alter the expression of **(A)** Representative dot blot, **(B)** Hsp70, and **(C)** Hsp90-β, or **(E)** TLR2. However, **(D)** 100 μM cocaine significantly reduced the expression of dectin-1 compared with the control condition. **P* < 0.05 indicates statistical significant difference between control vs. treated.

Supplementary Figure 5 Cocaine reduces HMC3 cell viability. HMC3 cells were incubated with 1 μM or 100 μM cocaine for 24 h to test the cell viability using MTT assay. Data indicated that cocaine significantly reduced HMC3 cell viability when compared with the control.

## Abbreviations

EV: extracellular vesicles
NTA: NanoSight Tracking Analysis
CD: cluster of differentiation
HSP: heat shock protein
TLR: Toll-like receptor.
