# Supplementary figures and images for: Effects of Cocaine on Human Glial-Derived Extracellular Vesicles
Source: Front Cell Dev Biol. 2021 Jan 11;8:563441. doi: 10.3389/fcell.2020.563441 (PMC7830252; doi:10.3389/fcell.2020.563441)

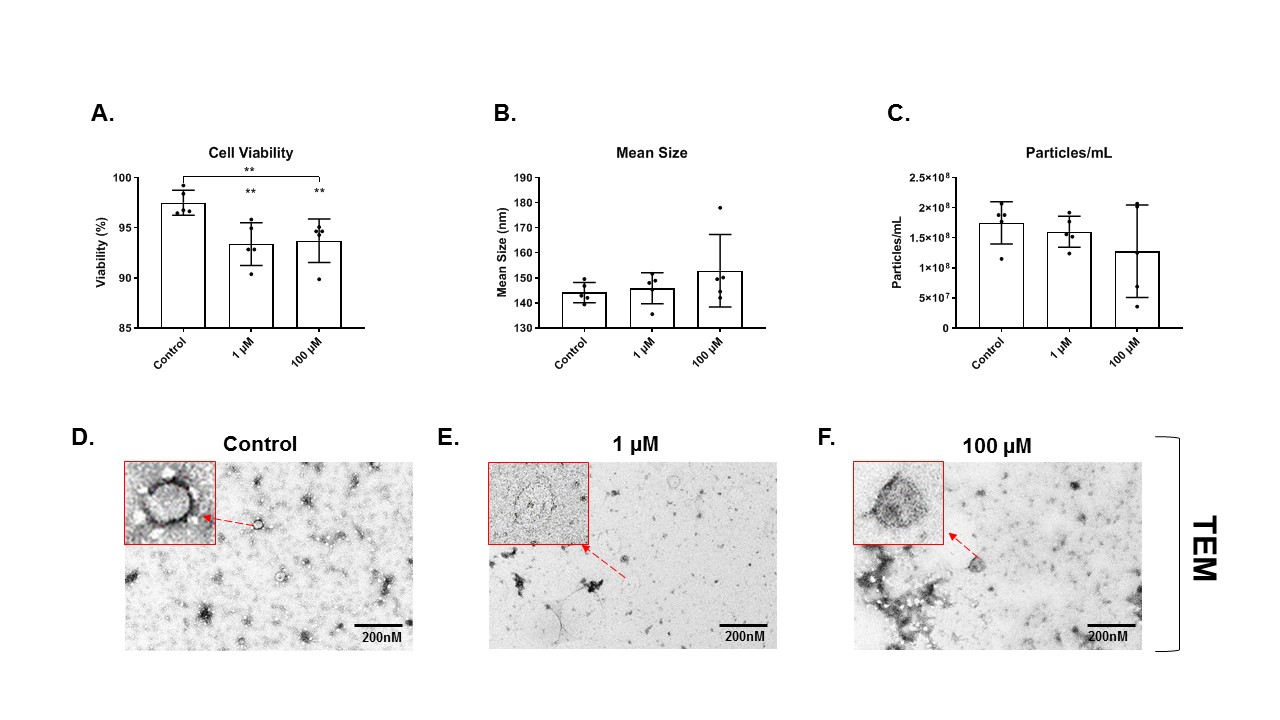

Supplement: Supplementary Figure 1 — Cocaine reduces HMC3 cell viability. HMC3 cells were exposed to 1 μM or 100 μM cocaine for 24 h, after which cell viability (trypan blue exclusion assay) and EV size and quantity were evaluated using NTA and TEM. (A) Cocaine significantly reduced HMC3 cell viability when compared with the control condition. Cocaine did not alter (B and D–F) EV size but (C) slightly decreased EV production (particles per mL) compared with the control condition, although this difference was not significant. Data were obtained from 3–5 independent experiments performed in triplicate. Statistical significance is indicated as *P < 0.05 and **P < 0.01. [file Image_1.JPEG]

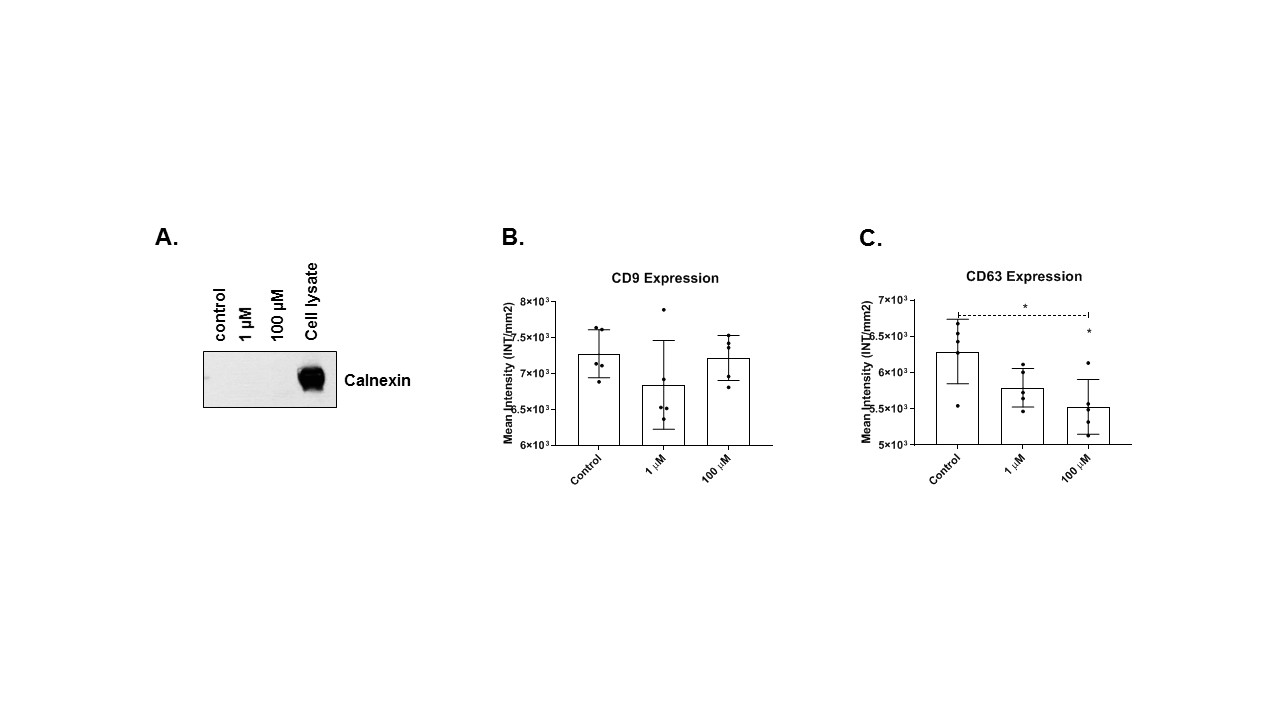

Supplement: Supplementary Figure 2 — Cocaine modulates expression of EV markers in HMC3 cells. Equal amounts (5 μg/dot) of total protein from HMC3 cell-derived EVs derived after treatment with 1 μM or 100 μM cocaine. These samples were loaded onto nitrocellulose membranes to assess the expression of CD9 and CD63. Cocaine (A) demonstrated purity of exosomes (B) did not alter CD9 expression but (C) significantly decreased CD63 expression compared with the control condition. Statistical significant difference obtained from 5-independent experiment is indicated as *P < 0.05 and **P < 0.01. [file Image_2.JPEG]

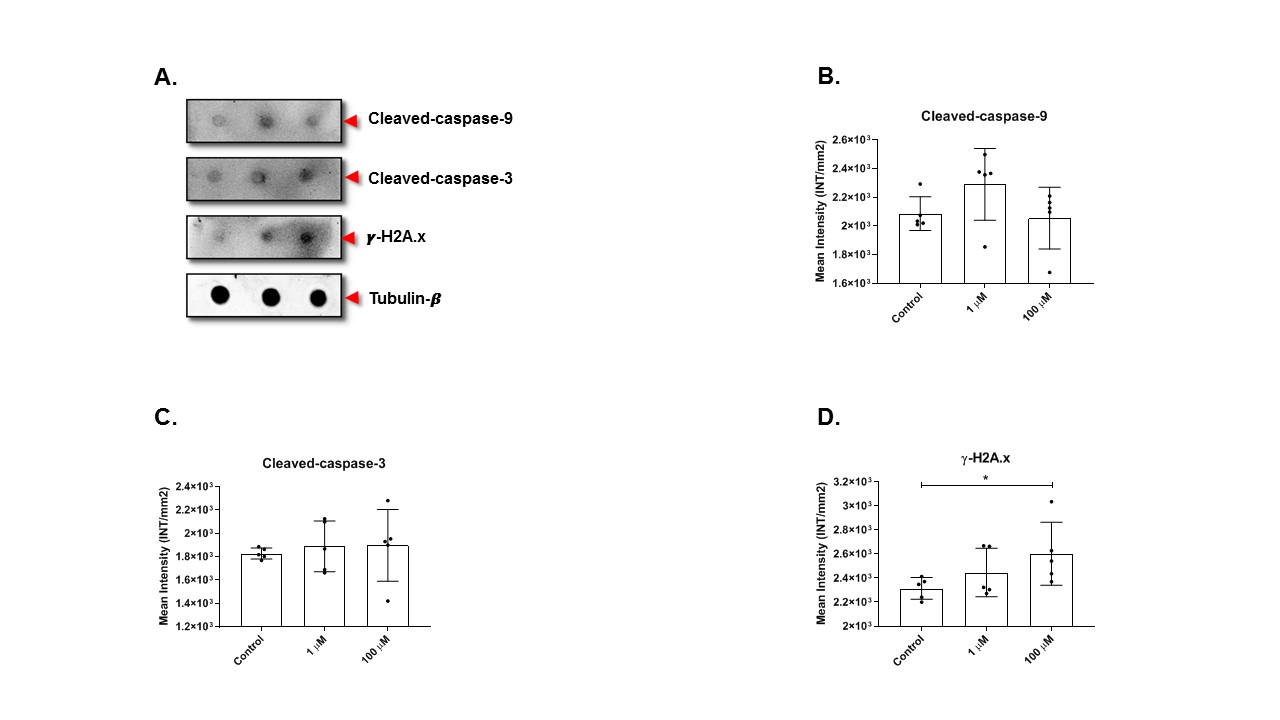

Supplement: Supplementary Figure 3 — Cocaine affects expression of apoptotic markers in HMC3 cell-derived EVs. (A) Equal amounts (5 μg/dot) of total protein from HMC3 cell-derived EVs derived after treatment with 1 μM or 100 μM cocaine. These samples were loaded onto nitrocellulose membranesto assess the expression of cleaved caspase-9/-3 and histone H2A.x. Cocaine did not alter the expression of (B) cleaved caspase-9 or (C) cleaved caspase-3. However, (D) 100 μM cocaine significantly reduced the expression of histone H2A.x compared with the control condition. Statistical values obtained from 5-independent experiment is indicated as *P < 0.05. [file Image_3.JPEG]

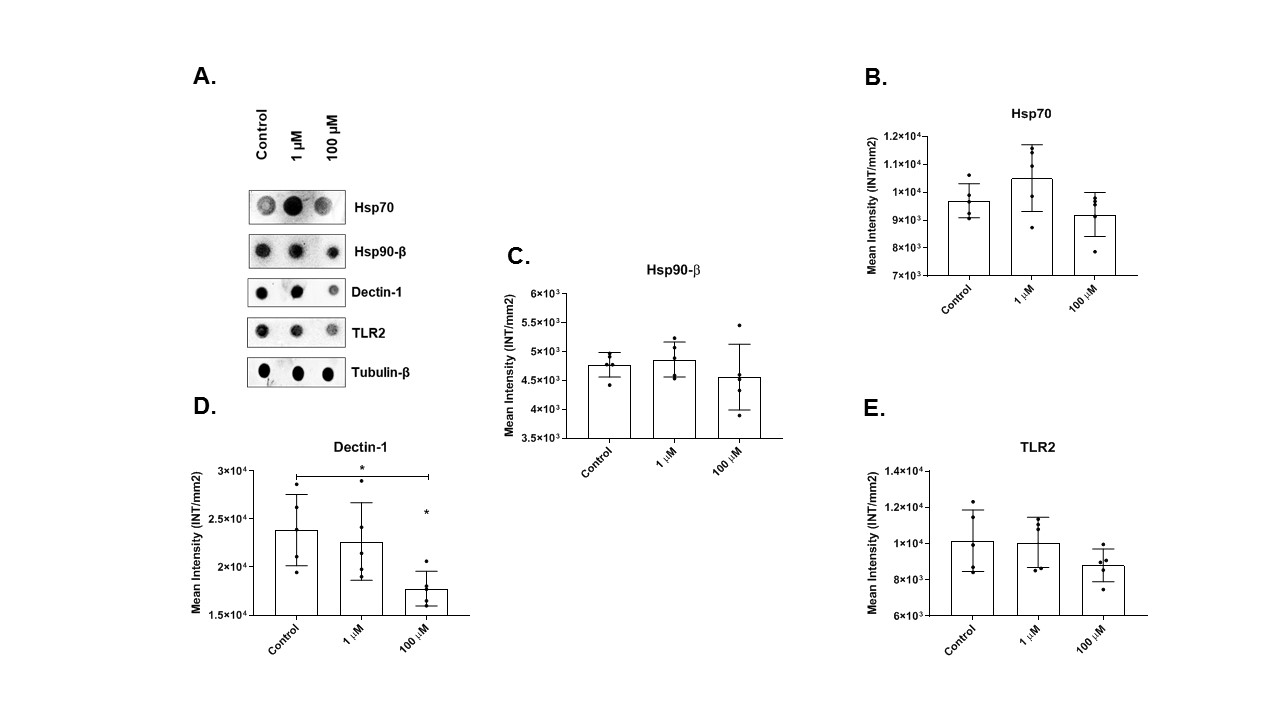

Supplement: Supplementary Figure 4 — Cocaine affects expression of dectin-1 in HMC3 cell-derived EVs. Equal amounts (5 μg/dot) of total protein from HMC3 cell-derived EVs were loaded onto nitrocellulose membranes. Cocaine did not alter the expression of (A) Representative dot blot, (B) Hsp70, and (C) Hsp90-β, or (E) TLR2. However, (D) 100 μM cocaine significantly reduced the expression of dectin-1 compared with the control condition. *P < 0.05 indicates statistical significant difference between control vs. treated. [file Image_4.JPEG]

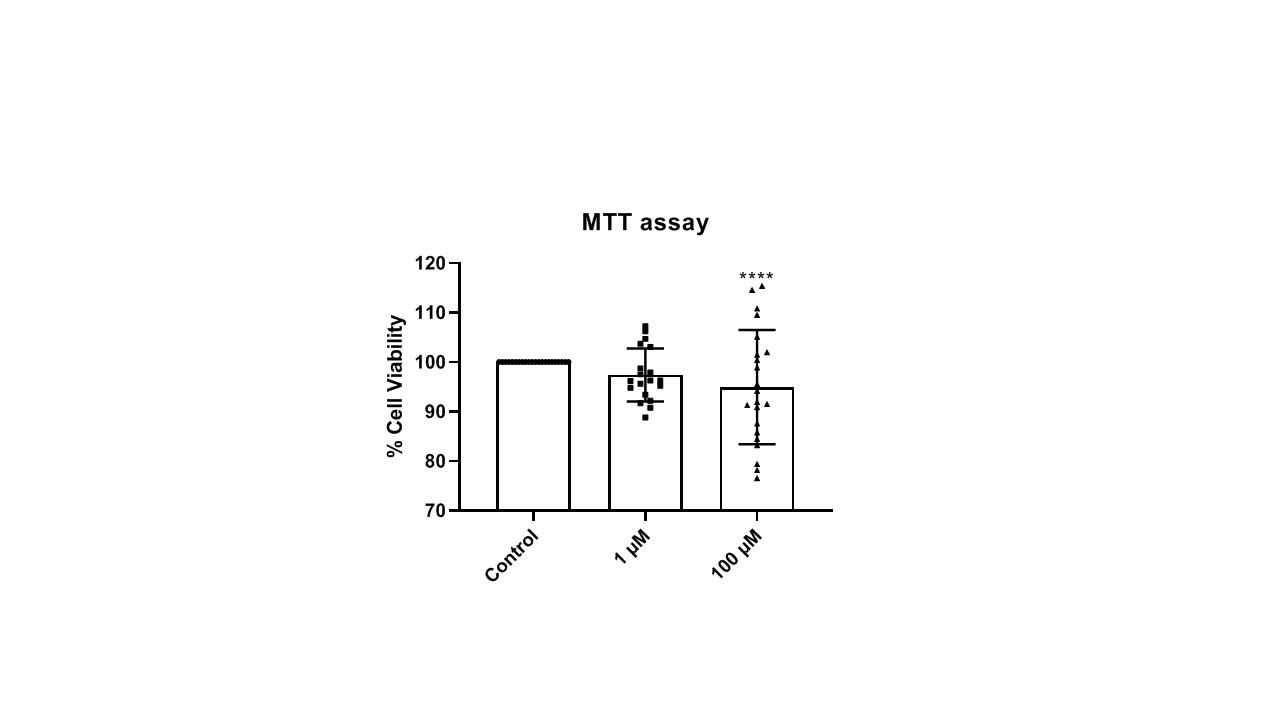

Supplement: Supplementary Figure 5 — Cocaine reduces HMC3 cell viability. HMC3 cells were incubated with 1 μM or 100 μM cocaine for 24 h to test the cell viability using MTT assay. Data indicated that cocaine significantly reduced HMC3 cell viability when compared with the control. [file Image_5.JPEG]
